# Postoperative photodynamic therapy as a new adjuvant treatment after robot-assisted salvage surgery of recurrent squamous cell carcinoma of the base of tongue

**DOI:** 10.1186/s12957-015-0630-6

**Published:** 2015-07-16

**Authors:** Vincent Vander Poorten, Jeroen Meulemans, Sandra Nuyts, Paul Clement, Robert Hermans, Esther Hauben, Pierre Delaere

**Affiliations:** Otorhinolaryngology-Head and Neck Surgery University Hospitals Leuven, Herestraat 49, 3000 Leuven, Belgium; Leuven Cancer Institute, University Hospitals Leuven, Leuven, Belgium; Department of Oncology, Section Head and Neck Oncology, KU Leuven, Leuven, Belgium; Radiation Oncology, University Hospitals Leuven, Leuven, Belgium; Department of Oncology, Section Radiation Oncology, KU Leuven, Leuven, Belgium; Medical Oncology, University Hospitals Leuven, Leuven, Belgium; Radiology, University Hospitals Leuven, Leuven, Belgium; Pathology, University Hospitals Leuven, Leuven, Belgium

**Keywords:** Oropharyngeal cancer, Photodynamic therapy, Transoral robotic surgery, Salvage surgery, Chemoradiation

## Abstract

**Background:**

For patients who remain with involved resection margins after transoral robot-assisted salvage surgery (TORS) for recurrent squamous cell carcinoma (SCC) at the base of tongue (BOT) following primary (chemo)radiotherapy, further adjuvant treatment options are very limited. We want to report on our preliminary experience with a new adjuvant strategy using postoperative temoporfin-mediated photodynamic therapy for this indication.

**Methods:**

Two patients with recurrent SCC after primary (chemo)radiotherapy of the BOT were treated with TORS, but unfortunately remained with involved resection margins on the postoperative pathology report. If left without additional treatment, these patients are prone to further recurrence. Temoporfin-mediated photodynamic therapy was used as a new adjuvant approach to treat the remaining microscopic disease at the resection margins.

**Results:**

Good oncological and functional results were obtained in these patients, now treated for a recurrence, after a preceding full course of radiotherapy. Both are disease free at 42 and 24 months of follow-up and are able to speak, breathe, and eat normally.

**Conclusions:**

In selected patients that have undergone salvage surgery with positive resection margins, postoperative temoporfin-mediated photodynamic therapy can result in a good oncological and functional outcome.

**Electronic supplementary material:**

The online version of this article (doi:10.1186/s12957-015-0630-6) contains supplementary material, which is available to authorized users.

## Background

Intensity-modulated radiotherapy (IMRT) with or without concomitant chemotherapy is now the standard of care for locoregionally advanced base of tongue squamous cell carcinoma (BOT-SCC) [1, 2]. “Resectable” locoregional recurrence following IMRT requires surgical salvage. This possibly implies a laryngectomy to avoid aspiration or a mandibulotomy for access and reconstruction. Recently, for selected patients, these open approaches have been replaced by transoral CO_2_ laser microsurgery [3] and transoral robotic surgery (TORS), [4] with better function and cosmesis. This postradiotherapy setting entails more difficult peroperative margin assessment than in primary surgery, and not infrequently involved resection margins on the definitive pathology report. Adjuvant treatment options to control this remaining microscopic disease are limited. It is often unclear where further meaningful surgery can be performed. Re-irradiation carries a low chance of disease control and additional toxicity. Temoporfin-mediated photodynamic therapy (PDT) has been described to effectively control recurrent SCC following radiotherapy [5, 6]. This is the first report of the use of PDT as a postoperative adjuvant treatment in patients with involved resection margins after TORS salvage surgery for post(chemo)radiotherapy recurrent BOT-SCC.

## Methods

Two patients with recurrent BOT-SCC after primary curative-intent treatment with radiotherapy (patient 1) or concomitant chemoradiotherapy (patient 2) were offered salvage TORS. Unfortunately, definitive pathology revealed involved resection margins. Both patients consented to postoperative PDT to address this issue. The institutional review board (Ethische Commissie UZ Leuven - Toetsingscommissie Klinisch Onderzoek) approved of a retrospective evaluation of their oncologic and functional outcome (IRB S56521).

### TORS

The Laryngeal Advanced Retractor System (LARS, Fentex®, Tuttlingen, Germany) and the Da Vinci S surgical robot (Intuitive Surgical® Inc, Sunnyvale, CA, USA), equipped with the 0-degree endoscope, a 5-mm Maryland dissector, and a 5-mm monopolar cauter spatula, were used to do the TORS procedure.

### Temoporfin-mediated PDT

Ninety-six hours following slow intravenous injection of Temoporfin (Foscan®) at a dose of 0.15 mg per kg, under general anesthesia, the surface of the resected tumor including a 1-cm margin was illuminated. We used a diode laser (652 nm wavelength) at a light dose of 20 J/cm^2^ and an intensity of 100 mW/cm^2^ through a microlens fiber (Biolitec, Jena, Germany). The target area was illuminated through a distendible laryngoscope (Karl Storz, Tuttlingen, Germany), shielding normal tissue around the target area. Postoperatively, patients made a gradual return to unrestricted indoor light exposure over a period of 2 weeks, with an increase of 100 Lux per day.

## Results

### Patient 1

In 2009, a 61-year-old male with a smoking history of 40 pack-years and a drinking record of 6 units of alcohol per day for 40 years, was diagnosed with an SCC metastatic right-sided cervical lymph node. Lacking primary tumor localization on positron emission tomography-computer tomography (PET-CT), a bilateral tonsillectomy and a right modified radical neck dissection were performed in a regional hospital. Three of 20 removed lymph nodes contained SCC, but no primary tumor was found. Postoperative radiotherapy was given to the nasopharynx, oropharynx, hypopharynx, and the bilateral neck (24 fractions of 2 Gray (Gy)) and a boost (6 fractions of 2 Gy) to the right neck.

**Fig. 1:**
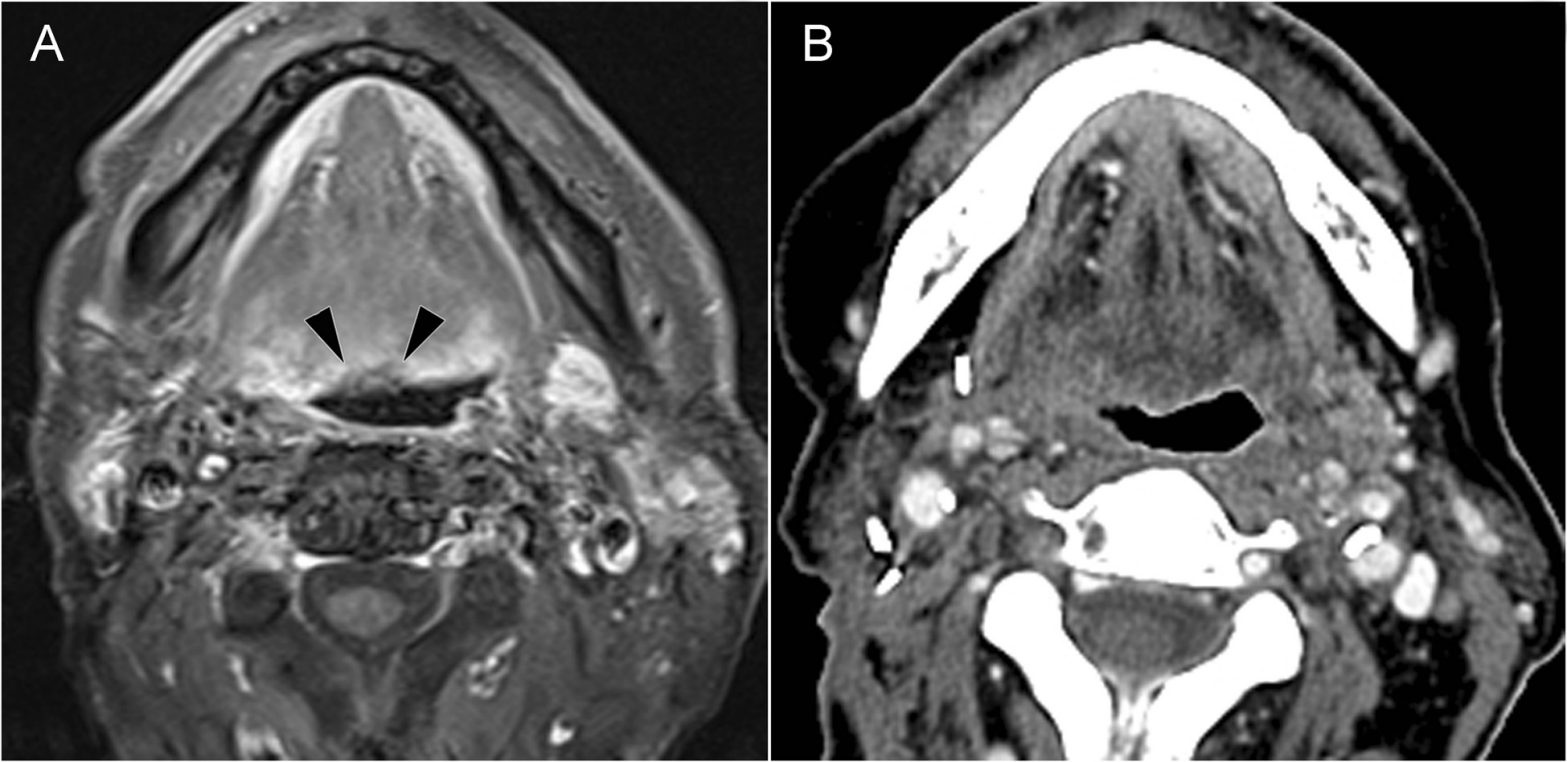
Patient 1. **a** Pretreatment axial contrast-enhanced fat-suppressed T1-weighed MR image shows small, irregular ulceration on the BOT midline. **b** Axial contrast-enhanced CT image: the BOT at 20 months post-TORS–post-PDT

One year later, he presented to us with a painful ulceration in the middle of the BOT. MRI confirmed a superficial ulceration. PET-CT excluded regional or distant metastases. A biopsy revealed a poorly differentiated SCC (stage rT1N0M0—Fig. 1a). The multidisciplinary treatment (MDT) board decided to offer salvage TORS for this recurrent BOT-SCC, during which there was satisfactory visualization of the tumor and macroscopically widely free resection margins. The patient resumed oral feeding on postoperative day 7 and left the hospital on day 8. The pathology report revealed poorly differentiated SCC with lymphovascular invasion. There was a widely negative deep margin but, unexpectedly, involved lateral resection margins due to scattered subepithelial lobular tumor nests. Redo surgery with expansion of the margins was judged to unlikely achieve free margins at a definite risk of considerable functional impairment. Discussing re-irradiation and temoporfin-mediated PDT as adjuvant options, the patient chose to undergo PDT. A tracheotomy was performed and the BOT was illuminated using four consecutive spots (diameter 2.5 cm—overlap 1 cm). Total energy delivered was 400 J.

Despite the negative pretreatment PET-CT, a CT of the neck, taken at day 12 following PDT because of an acute episode of pain, unexpectedly revealed an 8-mm centrally necrotic lymph node in zone Ib in the extensively operated and radiated neck. Needle aspiration biopsy proved SCC, so 17 days after PDT, the metastatic lymph node was removed. On that day 17, the nasogastric tube (NGT) was removed and on day 19, the tracheal cannula was removed and the patient discharged from the hospital. Since then, the patient speaks, eats, and drinks normally and remains disease-free at 42 months of follow-up (Fig. 1b).

### Patient 2

Patient 2 is a 64-year-old female, diagnosed in 2011 with a moderately differentiated SCC of the lingual epiglottis extending in the BOT and a right-sided metastatic lymph node (stage T2N1M0). Her medical history involved morbid obesity, arterial hypertension, type II diabetes, moderate renal insufficiency, 46 pack-years, and a history of 2 units of alcohol per day for 40 years. She was treated in a regional hospital with IMRT to a total dose of 70 Gy (50 Gy in fractions of 2 Gy with a boost of 20 Gy on the supraglottis and the cervical lymph nodes) combined with 2 cycles of Cisplatin. The third cycle was canceled because of neutropenia and progressive renal failure.

**Fig. 2:**
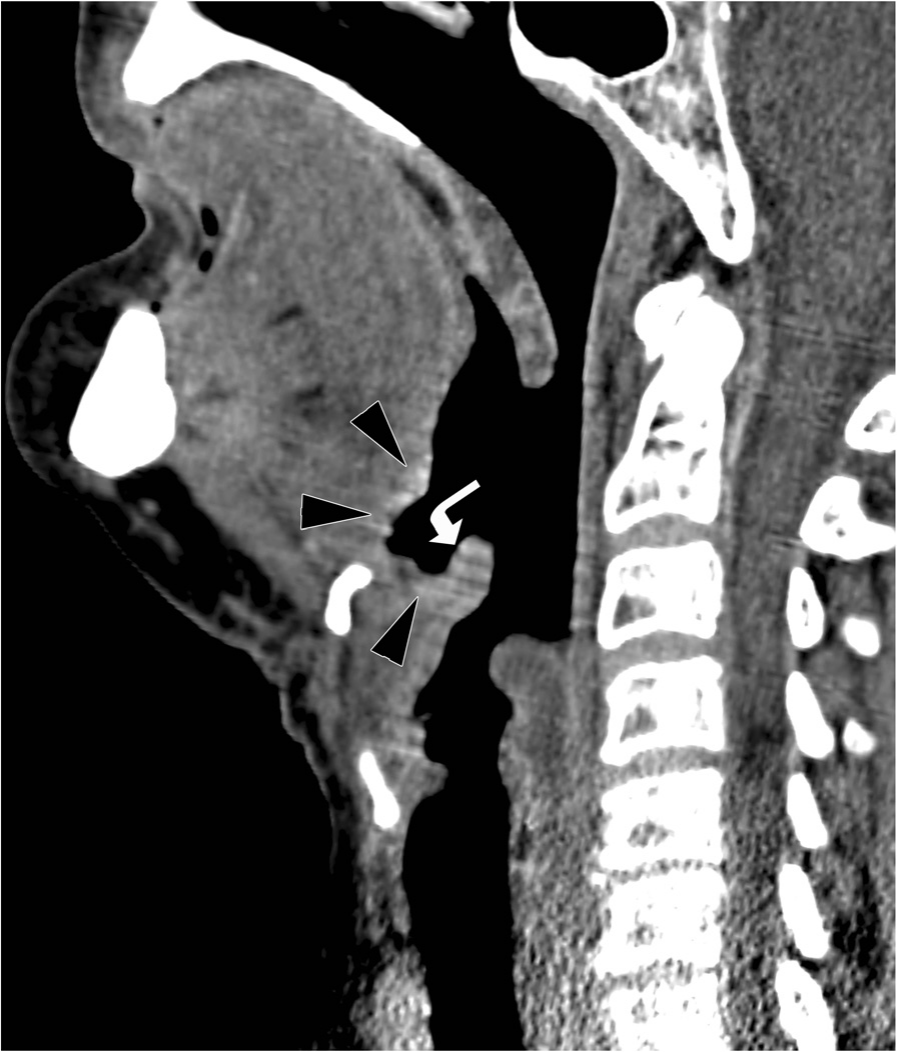
Patient 2. Pretreatment sagittal contrast-enhanced CT-image shows ulceration with marginal enhancement in the BOT midline (*arrowheads*); the soft tissue thickening extends on the lingual surface of the epiglottis (*curved arrow*)

Fourteen months later, a recurrent SCC in the right vallecula and lingual epiglottis with pre-epiglottic space extension was diagnosed (Fig. 2a). PET-CT additionally revealed a suspect right-sided level III lymph node. The MDT board decided to perform, in a first stage, TORS salvage supraglottic laryngectomy, including the vallecula, leaving the patient to recover from this surgery. In a second stage, it was planned to address the right-sided level III lymph node by a superselective neck dissection. TORS went well, with satisfactory visualization of the tumor and macroscopically widely free resection margins. Oral feeding was resumed 5 days postoperatively; the NGT was removed on day 15. The patient returned home on day 16, still tracheostomy dependent. Unfortunately, definitive pathology revealed a poorly differentiated SCC with a few very small tumor strands extending at different areas of the specimen into the margins. The MDT board concluded to associate postoperative adjuvant PDT of the primary site to the planned neck dissection. At 8 weeks, the superselective neck dissection (levels III and IV) revealed 1 metastatic node out of 17 removed lymph nodes. One week later, PDT of the BOT primary tumor bed, by one spot with a diameter of 3.0 cm to a total energy of 141 J, was performed. Postoperatively, the patient continued normal oral feeding. On post-PDT day 4, the tracheal cannula, in place since the TORS, could be removed and the patient was discharged home. At 24 months of follow-up, the patient is speaking, eating, and drinking normally and remains without disease activity (Additional file 1 Video 1).

## Discussion

In the salvage setting of recurrent BOT-SCC, the combination of TORS and adjuvant PDT for involved margins has not been previously described. Our preliminary experience in two consecutive patients with recurrent BOT-SCC following (chemo)radiation suggests that this approach can achieve locoregional tumor control, while preserving speech, respiration, and swallowing. In these patients, treated for a recurrence after a previous full course of radiotherapy, we left an interval between the surgery to the primary tumor and the neck to facilitate resumption of breathing and swallowing. We secured the airway using a tracheostomy, since both edema of the upper airway and aspiration can be expected after both TORS and PDT, especially in the postradiotherapy setting. For the same reason, it is safe to incorporate a 4- to 6-week recovery period after TORS, before proceeding to PDT. Expecting severe interference with the swallowing mechanism, NGT feeding is often necessary to ensure adequate caloric intake following TORS and PDT. The NGT could be removed 7 days after TORS and 19 days after PDT in patient 1 and 15 days after TORS in patient 2. In the latter patient, observing no important dysphagia after PDT, oral feeding was not interrupted. At hospital discharge after PDT, at 19 days in patient 1 and at 4 days in patient 2, both were able to eat, breathe, and speak normally. Follow-up visits were performed every 2 months for the first 2 years, and for patient 1, every 3 months in the third year and every 4 months in the fourth year of follow-up [7]. In patients with involved margins in a previously irradiated area, the observed oncological disease control, at 42 months for patient 1 and at 24 months for patient 2, would have been very unlikely without adjuvant PDT.

## Conclusions

Temoporfin-mediated PDT seems a meaningful adjuvant treatment with good oncological and functional results in selected patients, undergoing salvage TORS for recurrent BOT-SCC after primary (chemo)radiotherapy. The condition is that the microscopical remnant disease (R1 margin) can be fully illuminated during PDT. This approach merits further study concentrating on long-term locoregional control and functional outcome in larger groups of patients.

## Additional files

Additional file 1: Video 1. Indirect laryngoscopic view of the BOT, supraglottis, and glottis at 12 months after TORS and PDT of recurrent SCC in the right-sided vallecula and lingual epiglottis in patient 2.

## Abbreviations

BOT: base of tongue
BOT-SCC: base of tongue squamous cell carcinoma
CT: tomputer tomography
IMRT: intensity-modulated radiotherapy
IRB: institutional review board
LARS: Laryngeal Advanced Retractor System
MDT: multidisciplinary treatment
MRI: magnetic resonance imaging
NGT: nasogastric tube
PDT: photodynamic therapy
PET-CT: positron emission tomography–computer tomography
SCC: squamous cell carcinoma
TORS: transoral robot-assisted salvage surgery
